# “It’s my life, it’s my choice and I want to say when” vs “A good death is to be on good terms with God”. Comparing the views of people with dementia in the UK and Brazil about a good death: a cross-cultural qualitative study

**DOI:** 10.1186/s12904-025-01771-w

**Published:** 2025-05-16

**Authors:** Rasa Mikelyte, Karen Harrison Dening, Déborah Oliveira, Julia Maria Vanelli, Adriele Ferreira Neves, Natashe Lemos Dekker, Francelise Pivetta Roque, Edison Iglesias de Oliveira Vida

**Affiliations:** 1 Research Fellow, Centre for Health Services Studies, University of Kent, Canterbury, UK; 2 Head of Research and Publications, Dementia UK, London, England; 3 Researcher and Lecturer, Department of Psychiatry, Federal University of São Paulo, São Paulo, Brazil; 4 Universidade Andrés Bello, Faculty of Nursing, Campus Viña del Mar, Viña del Mar city, Chile; 5 Millenium Institute for Care Research (MICARE), Santiago, Chile; 6 Internal Medicine Department, Medical School Botucatu, São Paulo State University (UNESP), Botucatu, Brazil; 7 Neurology, Psychiatry, and Psychology Department, Medical School Botucatu, São Paulo State University (UNESP), Botucatu, Brazil; 8 Postdoctoral Researcher, Department of Anthropology, University of Amsterdam, Amsterdam, The Netherlands; 9 Associate Professor, Speech and Language Therapy Department, Nova Friburgo Health Institute, Universidade Federal Fluminense, Nova Friburgo, Brazil; 10 Professor, Internal Medicine Department, Medical School Botucatu, São Paulo State University (UNESP), Av. Mario Rubens Guimaraes Montenegro, SN, Botucatu, SP 18618-687 Brazil

**Keywords:** Dementia, Death, Palliative care, Cross-cultural comparisons, Qualitative research

## Abstract

**Background:**

It is unclear what People Living with Dementia (PLwD) consider a good death to entail, or how those perspectives vary according to culture and context. We aimed to compare the meaning of a good death for PLwD in Brazil and in the United Kingdom (UK).

**Methods:**

In this cross-sectional qualitative study, we conducted semi-structured interviews with a convenience sample of 32 PLwD (16 in Brazil and 16 in the UK) using jointly designed, equivalent interview guides. Two teams of interdisciplinary researchers independently analysed transcripts for their country using inductive thematic analysis, followed by jointly developing overarching themes on the contrasts and similarities across both settings.

**Results:**

We identified three shared themes: choice and control; spirituality; and fears and wishes. Choice and control permeated all aspects of what a good death meant to PLwD in the UK but was largely absent from Brazilian narratives. The opposite was true for spirituality, which was central to the meaning of a good death in Brazil, while far less prominent in the UK. In both countries, previous experiences with the death of others often shaped wishes and fears towards their own deaths.

**Conclusion:**

Our results have potential to expand the awareness and sensitivity of health and social care professionals around different cultural views on what a good death means for PLwD and what helps or hinders achieving it. Additionally, our findings challenge global indices of quality of death that do not take cultural and contextual differences into account.

**Supplementary Information:**

The online version contains supplementary material available at 10.1186/s12904-025-01771-w.

## Background

The circumstances in which people die have changed substantially over the last century and there is evidence of major gaps related to the relief of suffering at the end of life [1, 2]. To a large extent, palliative care emerged to address those gaps and make the experience of dying better [3]. Empirical studies on the concept of a good death from the perspective of patients and their families have been important for the development and maturing of the field of palliative care [4]. For example, in the United Kingdom (UK), cancer patients’ views on preferred place of care at the end of life has influenced the National Institute for Clinical Excellence Guidance on Palliative care for Cancer [5, 6].

Dementia is increasingly recognised as a life-limiting illness and its global prevalence is expected to rise from about 57 million cases in 2019 to 153 million cases in 2050 [7, 8]. The 2016 World Alzheimer Report concluded there is “an urgent need for more research, specific to the dementia field, regarding preferences of people with dementia” [9] and acknowledged the existence of a policy gap regarding end-of-life care for that population. A better understanding about the preferences of People Living with Dementia (PLwD) is particularly important because they are expected to lose the capacity to participate in shared decision making with the progression of their disease.

Who defines a good death with dementia is important. Professionals view a good death for people with dementia as one that prioritises comfort, dignity, and person-centered care, with a strong emphasis on pain management, familiar environments, effective communication and ensuring that end-of-life experiences are as peaceful and meaningful as possible for both the individual and their family [9–12]. Family members often focus on maintaining the personhood of the person with dementia, ensuring a peaceful death, and the importance of a preferred place for dying [11, 13]. Families also emphasise the need for pain and symptom management, clear decision-making, and ability to be present for their dying relative [14]. Very few studies focus on the perspectives of PLwD; only one out of 11 studies included in a recent scoping review on the definition of a good death for PLwD considered their views [11]. In that study, PLwD valued the acceptance of death, comfort, their physical appearance, the presence of family, solitude/being with God, the skill set of healthcare providers, honest communication, and a peaceful environment [15]. Another study directly comparing the views of PLwD and carers about end-of-life care found that although both groups valued remaining in their preferred place of care, minimising stress, and ensuring comfort, they disagreed on the importance of future care planning, responsibility for decision making, core competencies expected from healthcare professionals, and coordination of care [16].

Given the imbalance in whose perspectives on a good death with dementia are represented in research literature, more perspectives from people living with dementia are needed. However, this is not the only imbalance of perspective. Most research on the concept of a good death from the perspective of patients has been conducted in high-income countries, both in general (i.e. focusing on people living with cancer and other illnesses) and specifically in relation to dying with dementia [15–17]. The context in which research is conducted matters. For instance, in China, maintaining hope and not being a burden are emphasised when defining a good death, while in northern Tanzania, religious and spiritual wellness are key [18, 19]. Different ethnic and/or religious groups within the same country are also shown to construct a good death differently [20, 21]. To emphasise the need for ‘good death’ research that focuses on a variety of settings, a recent overview of systematic reviews on the conditions for a good death identified a need for research efforts aiming to understand how those conditions apply across cultural, religious and political boundaries [22]. Such research is particularly important because, without it, we risk generalising quality of death standards developed in affluent countries to large areas of the world where they may not be culturally appropriate. Importantly, that perspective is in line with the recent global decolonization movement, which acknowledges that health and care systems in low- and middle income countries have often been shaped by colonial perspectives, leading to disparities in health outcomes, and seeks to create more equitable and culturally sensitive health and care systems [23]. In addition, cross-cultural comparisons may reveal hidden patterns and assumptions by providing contrasting perspectives and may be instrumental in opening possibilities in supporting individuals and families to prepare for death, as well as to revisiting and addressing global challenges in palliative care [24].

The initial idea for this study was developed during a workshop between researchers in the UK and Brazil. EIOV, FPR, KHD and RM subsequently held a series of meetings to establish a research problem similarly relevant in both the UK and Brazil, where it was ascertained that palliative care research in both countries largely lacked the perspectives of people living with dementia. The rationale to compare countries instead of conducting two parallel, independent studies stemmed from the knowledge, discussed above, that research on the concept of a good death from the perspective of the patient/person living with a life-limiting illness, has predominantly been conducted and reflects perceptions of people in high-income countries, but tends to inform quality of death standards that are applied globally [17, 22, 25]. We wished to explore if perspectives on a good death with dementia are comparable across substantially different settings, in terms of societal and individual views on dementia, historical, as well as current state of Health and Social Care Provision in both countries, as well as religious, political, and wider cultural differences.

In short, our study aimed to compare the views of PLwD from the UK and Brazil about what a good death would mean for them.

## Methods

### Study Design

This cross-sectional qualitative study was conducted in the Southeast region of Brazil and in several parts of the UK. Our study design adheres to a social constructivist paradigm, which acknowledges the multiplicity of interpretations of what we call reality [26]. Such an approach is considered particularly relevant to illustrate how social constructs develop. Semi-structured interviews were conducted following a topic guide (Appendix 1). The interview guide was jointly designed in English by the research team members from both countries, and subsequently translated to Portuguese by the Brazilian team in an iterative process seeking harmonisation [27]. The topics were adapted from studies that focused on the experiences of people with cancer and their perceptions of a good death; topics were formed based on concepts found to be important in this previous work, such as preparing for death, comfort, presence of others at the point of death, place of death and others [28]. A person living with dementia provided comments on the interview guide, which was subsequently amended to reflect these. Moreover, all study materials (interview guide, consent form, participant information sheet) were reviewed by 3 members of a Patient and Public Involvement and Engagement Group in the UK. The Brazilian interview guide was further tested with 2 individuals without dementia to ensure that the English-to-Portuguese translation was optimal and easy to understand.

The eligibility criteria to participate in this study were having a diagnosis of dementia of any type made by a doctor, be aware of that diagnosis, and have capacity to consent for a qualitative interview. In both countries, participants’ capacity to consent was assessed in line with the principles outlined by Sudore [29].

### Context

Despite the fact that a comprehensive description of the Brazilian and UK culture and context lies beyond the scope of our study, we can provide several examples of characteristics of both countries that are relevant for our research. First, Brazil is a highly religious country and religion plays an important role in many aspects of Brazilian life, from family and community life to politics [30]. In contrast, the UK is a much more secular society where religion does not play a comparable role in the life of the population [31, 32]. Second, although both countries have universal health systems, the UK’s National Health Service (NHS) was created in 1948 with the promise of providing care from “the cradle to the grave”, whereas the Brazilian National Health System, that goes by the SUS acronym, was created in the 1990s. Most people in the UK, even the oldest-old, would not have a strong recollection of pre-NHS provision and see (health)care as a state duty to its population [33]. In Brazil, most older people still remember the pre-SUS provision era, when healthcare was not universal, and may have a different set of expectations from the healthcare system than people from the UK. Third, over the past decades, there were multiple campaigns to raise awareness about dementia in the UK, while any similar awareness efforts in Brazil have been fairly minor [34–36]. Fourth, in Brazil, it is common for multiple generations to live under one roof and families – particularly women – often take on the role of primary caregivers [37, 38]. In contrast, the UK has lower levels of intergenerational living and families may rely more on professional caregivers, domiciliary healthcare services, and long-term institutions to support their older relatives than taking charge of daily caregiving activities [39]. Indeed, in the UK, there is evidence that around one fifth of people diagnosed with dementia live alone at home [40]. In contrast, in Brazil, it is exceedingly rare for people with dementia to live alone [41]. Most of them live with, and are cared for by their families. Finally, in the UK, most people with dementia spend the end of their lives in care homes, whereas in Brazil less than 1% of the population of older adults lives in care homes and most people with dementia die in hospitals seconded by their homes [42–44].

### Recruitment

Participants in both countries were recruited using convenience sampling. In the UK, participants were recruited between August 2019 and June 2021; the first seven from dementia peer groups in East Kent, where the research was advertised, were interviewed at their residence, and the other nine participants were recruited from a Britain-wide dementia working group and via social media advert by a national dementia charity. In both instances, PLwD contacted the researcher to manifest their interest in participating in the study. The online interviews were conducted on Zoom in the UK and Google Meet in Brazil, using video as well as audio feeds to ensure the interviewee and the interviewer could see each other. Only audio recordings were used for data analysis; video was not retained. The researchers provided the participants or their carers with a weblink to the meetings. The in-person interviews were only audio recorded.

In Brazil, PLwD were referred for recruitment by health professionals (e.g., geriatricians, nurses, psychiatrists, and neurologists) from public and private sectors in the Southeast region of the country from September 2019 to August 2021. The research team then contacted the representatives of the PLwD by phone to make an invitation for the study. Seven interviews were conducted at their homes and nine were carried out online.

### Ethical considerations

Informed consent was taken either in writing, or verbally (the latter audio-recorded for evidence); we checked for ongoing consent throughout the interview [45]. After, participants were interviewed and asked about their demographic characteristics and dementia diagnosis.

We have carefully considered the potential implications of interviewing individuals living with dementia about death and dying, and approached both the design of the topic guide, the interviewing process and the capacity-assessment process with sensitivity. At the start of the interviews, the interviewers acknowledged that this may be a difficult topic, and reassured the participant that it would be entirely acceptable to pause or discontinue the interview, take a break, as well as to not answer questions they do not wish to answer. A distress protocol for research on sensitive topics was used to identify, assess and manage distress during the interview process. While some participants cried during the interview, particularly when talking about their loved ones, all have chosen to continue the interview.

This study was conducted in agreement with the principles outlined in the Declaration of Helsinki. Ethics approval was granted by Botucatu Medical School (12,370,419.4.0000.5411) on May 16, 2019, and by the University of Kent Research Ethics Committee (SRCEA222) on March 18, 2019.

### Data collection and analysis

All interviews in the UK were arranged and carried out by a female social care researcher (RM). In Brazil, interviews were conducted by a male geriatrician (EIOV), a female psychiatrist (AFN), and a female medical student trained in qualitative research (JMV). Interviews were conducted in Portuguese in Brazil, and English in the UK; all participants and interviewers were fluent in the language in which the interview was conducted. Researchers followed a reflexive approach to interviewing [46]. Participants did not have previous relationships with the interviewers.

All interviews were transcribed verbatim and analysed thematically using the Taguette software [47]. Similarly to Nvivo software, often used in high-income countries, Taguette allows to highlight and tag words/sentences/paragraphs and create custom codes, access all highlights for the same code in a single space regardless of how many separate transcript files they originate from, and to combine/merge codes into higher-order codes. Nvivo could not be used as Brazilian universities do not routinely subscribe to Nvivo licenses the way UK institutions do; Taguette is a free, open-source tool designed to address inequities in access to data analysis software. Field notes were made after the interviews whenever appropriate.

Firstly, independent inductive thematic analyses were performed by authors from each country (RM and KHD in the UK, analysing transcripts in English, and EIOV, FPR, DO, and NLD in Brazil, analysing transcripts in Portuguese) [48]. Inductive thematic coding involved researchers reading and interpreting the text, highlighting part/whole sentences or paragraphs and creating codes which summarised what the interviewee was talking about [49]. In each country, a minimum of two researchers initially coded the same transcripts in parallel, then met to discuss, refine and standardise coding approaches. Afterwards, transcripts were split between coders for individual coding. After all transcripts in each country were coded, researchers met again to group similar codes into themes, and refine these themes. We chose thematic analysis because of the shared experience of researchers from both teams with this approach, its theoretical flexibility, and its suitability to examine the perspectives of different research participants, highlighting similarities and differences. The personal and professional background of the authors involved with the data analysis is presented in Appendix 2. The diversity of perspectives of the authors and the multiple dialogic iterations that characterised the process of theme generation contributed to the enrichment of our study. All six authors involved in country-specific analyses then followed this up by joint iterative discussions of the independently derived codes and themes. Each team ceased data collection when its understanding of the research questions reached a satisfactory level of complexity according to the concept of information power, i.e., when the collected data were rich and diverse enough to provide nuanced insights and sufficiently address the study’s objectives without requiring further data to enhance meaning or interpretation [50]. We then designed a new combined thematic framework representative of both datasets, focusing on the commonalities and differences between countries. Finally, we contrasted the relationships between themes for the two countries. Verbal consensus was sought during coding and analysis, revisiting data until agreement was reached, in line with existing approaches to qualitative analysis [51].

To preserve anonymity, participants were given an identifying code; for example, BR06 refers to participant 6 in Brazil, while UK15 refers to participant 15 in the UK. In preparing this report, we followed the Standards for Reporting Qualitative Research (SRQR) and the COnsolidated criteria for REporting Qualitative research (COREQ) checklists (Appendixes 3 and 4, respectively) [52, 53].

## Results

We interviewed a convenience sample of 32 PLwD (16 in Brazil and 16 in the UK), all of whom were cognitively able to provide informed consent. One UK participant lived in a dementia village and one ﻿Brazilian participant lived in the home of a paid carer [54]. All other participants in both countries lived at home. In the UK, where 22 people expressed an interest in the study initially and agreed to receive more information, 6 either declined to participate or did not respond to follow-up contacts. In Brazil, the corresponding figure was 4 people dropping out from 20 who were initially interested in hearing more information about the study. In the UK, all participants who scheduled interviews were able to provide informed consent and none were excluded based on capacity assessment. In Brazil, 3 individuals were excluded because they were not able to provide informed consent. All prospective participants in the UK sample were able to give informed consent and therefore none were excluded. While all participants in Brazil had a carer present during the interview, only 3 participants in the UK had a partner present based on their own preference. All participants were interviewed a single time. The average length of the interviews in the UK and Brazil was 59 (range 32 to 92) and 56 (range 30 to 93) minutes, respectively.

Table 1 describes the characteristics of participants from both countries. We were able to interview PLwD with a range of different characteristics in terms of age, gender, education, religion, time since diagnosis, relationship with the main carer, and living arrangements. Only in Brazil, we managed to include participants with self-reported race other than White.

**Table 1:** Characteristics of participants in Brazil and the UK

	**Brazil** **(*N* = 16)**	**UK** **(*N* = 16)**
**Gender**		
Female	11	12
Male	5	4
**Age,** median (min, max), years	80 (68, 90)	62 (49, 76)
**Race**		
Black	3	0
Brown (mixed race)	1	0
White	12	15
Not informed	0	1
**Time since diagnosis**	2y (1 m, 5 y)	5y (3y, 14y)
**Education**		
Primary school	11	1
Secondary school	2	4
Vocational school	2	5
University		5
Postgraduation studies	1	0
Not informed	0	1
**Religion**		
Catholic	12	2
Christian not otherwise specified	1	2
Church of England	0	2
Church of Ireland	0	1
Spiritism/Spiritualism	2	2
Evangelicalism	1	0
Humanist	0	1
Jewish	0	1
None	0	5
**Type of dementia**		
Alzheimer’s	13	6
Lewy body dementia	2	0
Vascular Dementia	1	1
Mixed dementia	0	4
Posterior Cortical Atrophy	0	3
Not known	0	2
**Relationship with main carer**		
Spouse	4	7
Daughter	8	1
Spouse and daugher	0	1
Niece	1	0
Mother	0	1
Sister	1	0
Professional carer	2	0
None	0	6
**Lives with carer**		
Yes	15	9
No, lives alone	1	6
Not informed	0	1
**Carer present during interview**	16	3

*m* month, *y* year, *min* minimum, *max* maximum

Three themes were identified: choice and control, spirituality, and fears and wishes, described and compared as follows.

### Choice and control

The choice and control theme covered: 1) the importance placed on choice and control in relation to a good death; 2) the function of choice and control in relation to death and dying; 3) tensions around maintaining control; and 4) expression/forms of feeling in control.

Choice and control was the first theme to demonstrate a clear divergence between countries, both in terms of level of importance and the function it served. Choice and control was a central theme for the UK interviewees, and permeated all aspects of a good death in this particular context.

*
“It’s my life, it’s my choice and I want to say when.” (UK01)*

In contrast, for the majority of Brazilians, choice and control was not even considered conceivable in relation to death.

*“Oh, I think that I, it isn’t, how to [have] control [over our deaths]. I think that when it happens, it happens, and it’s done. I don’t think there is any way to control death.” (BR7)*

In the UK, talking about choice and control appeared to be a way to deal with uncertainty about the end of life with dementia and to anticipate a time when they may no longer be in control.

*“I would think the preparations I’ve made is that, erm, there’s a time when you can’t… you won’t be able to voice what you want, that I’ve had the conversations. So that is part of my control, of having the conversations beforehand, putting the legal things in preparation beforehand. Doing all I can to make it easy for the people looking after me, both professionally and not professionally” (UK15)*

In contrast, the negative feelings brought by uncertainty were alleviated by spirituality in Brazil, rather than by the desire for having choice and control, as will be addressed under the next theme.

*“Look, I just say what follows: what God… thought to do with me [laughs], He will do [it] and [it’s] fine. Everything must happen as God commands […] we are here on earth because he brought us here” (BR3)*

In the UK, choice and control were so important that participants even considered compromising where they die and who they have present, if they could retain control about their death (i.e., seek euthanasia elsewhere). It is important to note, however, that euthanasia or assisted dying were not mentioned by the interviewers in any form (i.e., not part of the interview guide).

*“I really wish, wish to die when I want to die, if dementia starts to get hold of me, which in this country euthanasia isn't allowed, you know. I'd have to go to Switzerland or somewhere. And I wish that before I need that decision to be made, that the laws here will change. Because that would be a good death for me to decide whether or not.” (UK09)*

In Brazil euthanasia is also illegal, but unlike in the UK, there were no participants mentioning euthanasia and assisted dying. Moreover, when asked by the interviewer what control of any form would feel like, participants often seemed puzzled by the question. They struggled to think—what form of control would be possible when nearing death, but also found prospective thinking as an act of ‘anticipating’ their own deaths, which should be avoided.

*“No, I wouldn’t like [to know what may happen when I’m dying], because I would, would be anticipating my death if I tried to know. No, I wouldn’t like [to have control over my death]. Because, we have, have to let death the way God wants it to be, right?” (BR09)*

### Spirituality

The spirituality theme encompassed: 1) the importance of God/religion in relation to a good death; 2) the function/influence of spirituality; 3) religion as a source of comfort/concern.

Even though the majority of people in both countries disclosed having some religious affiliation, spirituality (used here as inclusive of religiosity, but also encompassing a sense of purpose, inner peace, and connection to a higher power or the universe) appeared to vary in role and significance across both countries [55]. In Brazil, these were major drivers of participants’ views around death, with some even framing a good death as “being at peace with God”. From that perspective, having faith in God also meant not questioning God’s will/leaving control with God.

*“A good death is to be on good terms with God. [...] [And to be on good terms with God] is to have faith in Him, right?”* (BR10)

In the UK, spirituality and religion were less central in participants’ lives and not a main lens through which death and dying were understood, even for those with a religious affiliation.

*“I don’t know whether having a faith helps you or not.”* (UK14)

For some Brazilian participants, their spiritual beliefs were associated with a certainty that nothing bad would happen during their death because they had absolute trust that God would protect them, and therefore it was not necessary to think about preparing for death.

*“God prepares everything for us. I have a huge faith. I am certain that I will have a peaceful death! I’m not afraid of dying! It is a crossing that we all must face! (…) I don’t think about death. I know that I will have a wonderful death!”* (BR1)

In contrast, in the UK, the role of God was resigned more to the afterlife rather than the period approaching death, where participants saw ensuring a good death as a matter of their responsibility.

*“Give us different choices [...] I like the smell of incense and I have mass online, on YouTube in my playlist and things like that [...] maybe God will be kind and [...] he’ll just say, ‘[name], you're going to have a pleasant time here and you don't need to fight anymore. Just let the world take care of itself. This is your ending. You know where you're going’”.* (UK15)

Rather than drawing comfort from religion, a number of UK participants expressed concern about the role of religion around death and dying and how religious beliefs of others may impact their wishes being respected. Such views were entirely absent in Brazil.

*“I absolutely want to live as positively as possible for as long as possible. But, just as I don’t see why, due to anybody’s political or religious beliefs that I should be enforced to live beyond what I consider is a good standard of living.”* (UK08)

As previously explored under the ‘choice and control’ theme, Brazilian participants gained comfort in the idea that God was in charge of defining the time and circumstances of their death. This was so reassuring for them that even having some experiences of pain or other forms of distress was seen as acceptable because it was seen as God’s will, which, by definition, is always kind.

*“Oh, I have wished for a good death. I have not wished for a painful death, right? If it [a painful death] happened, if it happened... the death that God has marked for me, I accept it, but I want a good death. But if, in that hour, whatever God sends me will be fine.”* (BR9)

Conversely, in the UK, ‘the will of God’ was a much less accepted concept. For example, one participant recounted being told by a friend that their dementia and cancer diagnoses was imposed by God:

*“I’ve heard some funny things […] because… In 2017 I got diagnosed with dementia, 2018 I got diagnosed with bladder cancer… And he says to me [...] that’s God’s way of cleansing you. He says er, it’s cleansing you of all your sins to get you prepared for the end of life. [laughs] And then I thought: hold on, if I’ve got dementia and cancer, I must’ve committed a lot of sins! [...I]n his religion that’s the way they see it [...] it’s getting rid of the sin, it’s getting rid of the punishment, it’s a punishment for anything you’ve done before your final journey.”* (UK12)

Religiosity was also present for several Brazilian participants who wished, for example, to receive a prayer at the time of their death, have the presence of a priest, or to participate in religious rituals.

*“Sure, I would like [to have a priest by my side in the last hours of my life]. I am a Roman Apostolic Catholic. [...] I wish he [the priest] would give me his blessing. [...] Because it would seal, it would seal everything in my life. [...] What I was in my life, for me and my family. [...] Because the priest is in touch with God… and that contact with God, for me, is above [everything]”* (BR14)

In the UK, some participants expressed that, rather than being helpful or reassuring, religious beliefs and practices of family members and professionals may instead deprive them of choice, should their preferred way of dying clash with others’ religious principles.

*“Because it’s my death, not yours, and it’s my wishes, you know. And no matter... and leave your religion and your thoughts and your feelings behind and just respect my beliefs at that moment” (UK15)*

### Fears and wishes

The fears and wishes theme comprised the following: 1) the influence of deaths of others on fears and wishes; 2) the influence of a dementia diagnosis on fears and wishes, 3) the fear of un(der)treated symptoms and neglect; 4) the fear of loss of self; and 5) fears/wishes around impact on others.

Fears and wishes around death and dying with dementia presented some similarity between Brazil and the UK. In both countries, participants (often strongly) framed their death-related fears and wishes based on previous experiences with the death of others, as well as both historical and current state of health and social care provision around deaths witnessed.

“*[A good death] is that [we] don’t suffer when dying. How do we say it? Dying quietly [laughs]. [...] Not suffering. [...] Feeling pain, like my mother [who] suffered to die, poor her, she suffered so much. [...] I think that death is suffering [...] because she stays in that agony to die, it took my mother a long time to die, but [she] stayed in that agony trying to talk without being able. It was like that.*” (BR15)

*“[M]y granddad had dementia [...] and then he died, you know, being quite agitated because he didn't really know where he was... [...] he died quite peacefully, but the lead up to the death I thought was quite complex for him, agitated, being in a strange place, not understanding where he was - that's not a good thing. [...] If they encounter me and I say I have end-stage dementia and I'm rather confused [...] you don’t want to be agitated, you want it to be peaceful. That's how it should be”* (UK05)

Regardless of whether respondents lived in the UK or in Brazil, most feared dying in pain, experiencing distressing symptoms and environments, a prolonged period of dependency/confinement to a bed, and neglect/abandonment.

*“I don't want to go down that road... it frightens me! That does more than death. I just don't frighten now... I'm not scared of death - death is my friend so to speak but becoming... what's the old word they used - senile. Becoming incontinent, having to be fed [...] that sort of stage really frightens me. That really does. But as for the final curtain, so to speak - no.” (UK05)*

*“I’m afraid of being sick [...] being dependent on others.” (BR5)*

Fears for others, particularly family, were also present in both settings. Participants were worried about being a source of ‘suffering’ or ‘burden’ to their families to such an extent that in both countries there were participants willing to forgo their wishes to die at home or to have family members close by at the time of death, in an attempt to protect their loved ones.

*“I don't have a partner… and my children - I don't want them to have the full burden of being there for me.”* (UK10)

*“I never thought about being afraid of something, but I wouldn’t like that my children saw my departure. I wouldn’t like it because I saw my mother’s and it was very hard, just that… I want them [my children] by my side while I’m suffering, but at the moment when I deliver my spirit to God, I would like to be just me and God.”* (BR11)

Despite the similarities in the main fears and concerns, there were also some important differences regarding specific fears between the two countries. One of the most striking differences involved the fear of losing one’s sense of identity. This kind of fear was only present in UK interviewees.

*“I don’t want to go into the stage where I am no longer me if you like…I am just an empty shell, that’s operating …you know…and I am not aware of…”* (UK01)

In Brazil, when participants appeared to recognise what the natural course of dementia would entail for their cognitive and relational abilities, they did not speak of it in such terms, i.e., as if they were losing their sense of self. Sometimes participants spoke about the course of dementia as becoming ‘childlike’ or by stating that dementia"is not going to change what [they] think or who [they] are"(BR16). The changes associated with the progression of dementia were seen as inevitable, thus as something that was not worth worrying about.

*“Ah, then we end up losing sense, right? Ah [laughs], then [we] become like a child, but [it] is… all that we must go through in life, right? There is nowhere to hide, right? Yes, we… What will happen to us… I think it is something that is going to happen…” (BR06)*

Another fear that was specific to UK participants involved the expectation that the health and social care systems would not be able to provide the support they needed throughout their illness trajectory. To a large extent, UK participants placed the responsibility for their future care in the health and social care system and a bad death was often blamed on the failure of professionals or the care system as a whole.

*“I have been left vulnerable, through lack of local knowledge. And lack of someone to talk to that has the time, and it’s their job. It’s their space. Your GP, they’re supposed to, but they don’t have the time.” (UK15)*

In Brazil there was a near-complete absence of any comments regarding expectations from the health and social care systems. Instead, participants placed the responsibility for their care on their families and seemed to believe that the circumstances of their death were largely determined by the will of God, as described previously.

*“[Talking about a bad death] I have seen people who had a family but lived alone, kind of abandoned. When people came to visit them, sometimes they were found dead. [...] Yeh, [he] died abandoned by family because there was no family to care for him. [On one day] someone would come to care for him, on the other day someone else would come to help.” (BR09)*

The divergent perceptions created notable differences between UK and Brazil cohorts regarding how participants perceived their diagnosis of dementia to influence the quality of their death. In the UK the perception of losing one’s identity, the erosion of the ability to exercise control over one’s death, and the uncertainties of a future with dementia within an unreliable health and social care system, were sometimes associated with wishes to make decisions in advance, sharing their care preferences with their families or through advance directives, and even a wish for euthanasia.

*“It would mean, actually, that I'd have to make the decision before I'm actually ready. Before I go over the edge into someone I don't recognise [...] If I didn't have dementia, a good death would look very different. It would.[...] dementia has taught me that … I will only be in control for so long. And it's when we become in the control of others that I don't want to experience. So that's why I brought my death forward, if you like, to still be me … to be in control of my life and my death” (UK09)*

On the other hand, in Brazil, the lack of these specific fears of loss of self and lower expectations from health and social care systems, appeared to buffer that feeling of uncertainty and were associated with the wish to focus on the present moment of life and avoid thinking about or preparing for their end of life. Hence, for Brazilian participants it did not matter if they would die from dementia or, for instance, heart failure.

*“A person who has Alzheimer’s disease is not different from others. For me there is no difference! […] I don’t think about Alzheimer’s. I keep going on with my life. [...] I don’t keep thinking about death, no. I want… hum… to lead the life that I’m leading. I do only what I want. [...] I don’t think about death. I know I will have a wonderful death! [...] Because I ask God [for it]” (BR01)*

### Relationships between themes

**Fig. 1:**
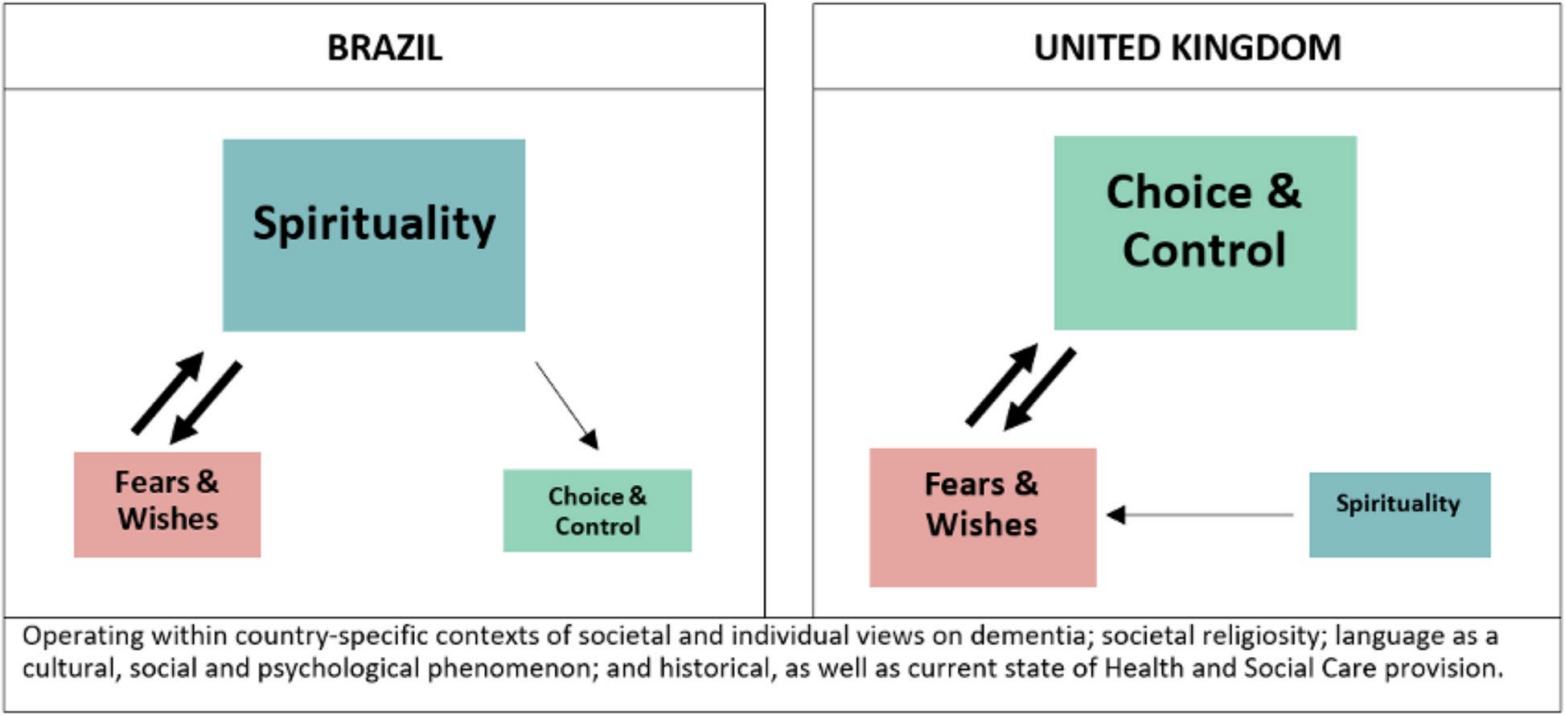
Relationship between themes from Brazil and the UK with regards to a good death in dementia

When considering the relationships between themes as well as comparing these between the two countries, we identified several overlapping features (Fig. 1). First, in the UK, fears and wishes were strongly connected to choice and control in a bi-directional manner. Fears could be alleviated by exercising choice/control, and a perceived lack of control was a source of fear. Second, in Brazil, choice and control was not often thought of as an option for participants, likely because it was not a way by which they alleviated their fears or expressed wishes. On the other hand, in Brazil spiritual/religious beliefs were connected in a bi-directional manner with fears and wishes. At the same time that spirituality served a protective function for Brazilian participants as a means to assuage any momentary concerns/fears related to their deaths, several wishes for their last hours of life were shaped by their religiosity. Such a relationship between fears and spirituality was absent in the UK even for participants who had a religious affiliation; instead, religious beliefs of others, such as health and care professionals, as well as family members, were often a source of fear that one's own wishes will not be respected. Lastly, in Brazil, the importance of spiritual/religious beliefs influenced participants’ unwillingness to consider choice and control around their deaths.

## Discussion

To our knowledge, this is the first study to directly compare the views of PLwD regarding a good death across two countries, and the first ever conducted with such aim in Latin America. By investigating the concept of a good death for PLwD, we acquired a better understanding of how different cultural settings shape death and dying experiences. Our findings show that, for PLwD in the UK, a good death often revolves around the notion of choice and control. For UK participants, having as much choice and control as possible at the end of life was not only a means to achieve a good death, but a goal in itself, where having control equated to having value as a person. Contrastingly, for the Brazilian participants, the idea of a good death was centred around spirituality, which was considered not only a means to achieve peace at the end of life, but a major component of a dignified death.

This broadly fits with findings from non-comparative studies on a good death that do not focus on dementia. In predominantly religious cultures, spirituality has been demonstrated to be a crucial component of quality of life and, by extent, the perception of a good death [55]. A non-Western perspective on a good death often follows religious rites and rituals, and the wider concept of spiritual experiences [56]. Conversely, in Western societies, where individual autonomy is emphasised, control over the dying process and the ability to make decisions about end-of-life care is more common [57]. The relatively lower importance placed on choice and control in non-Western settings is implied, but shared decision-making and preparing for death have hitherto been suggested as universal [17, 58]. Our comparison, where Brazilian participants rarely wished to exercise or express choice and control is particularly novel and the first to show that in some contexts control is undesirable. This goes further to challenge the centrality of making decisions about end-of-life care in definitions of good palliative care.

It is also noteworthy to compare how our findings on a good death with dementia compare to broader findings from related studies in each country. While research is limited, many Brazilians with incurable cancer seem to wish to die at home and in the presence of family; while the importance of family was pronounced in our study, too, a wish to die at home was not as strongly presented in our data [59]. The same Brazilian study of cancer patients has also shown that type of cancer and type of treatment received influenced some participants’ preference to die in a hospital; our study did not observe any trends in preference that depended on the participant’s type of dementia (in either Brazil or UK). Another Brazilian study of cancer patients in hospital witnessing the death of others suggested that a good death was seen as a ‘peaceful’ one [60]. We also found a peaceful death to be an important aspect of a good death in our Brazilian sample of PLwD and qualified it further by showing that being on good terms with God was a major component of being in peace. A greater number of studies on patient perceptions of a good death have been conducted in the UK, although these also focus predominantly on cancer patients. As is echoed in our findings, being free of pain and other distressing symptoms was regarded as crucial for a good death. [17, 58] Related to the importance of choice and control in our study, other studies have also found the perception of a good death to be related to involvement in decision-making in order to reduce anxiety and feel respected [61]. UK-based studies also show that control over treatment decisions and other aspects of death is more pronounced with some patient populations, for example patients with AIDS [62]. This underscores that the meaning of a good death may in part depend on the life-limiting illness and provides further justification for research on specific conditions, such as dementia.

Our study does just that, providing a dementia-specific direct comparison of perceptions of people living with dementia in the UK and Brazil. It showcases that the considerable, sometimes near-dichotomous differences in what is and what is not important for a good death in dementia in Brazil and the UK is not merely a result of different methodologies applied or questions asked. The only part-qualitative study looking at perceptions of PLwD in Long-Term Care in Canada showed similar findings to our UK sample, namely that good end-of-life care included being treated with respect, being involved in care decisions, and having basic needs met [15]. This is crucial when quality of death and quality of care at the end of life measures are applied globally. For example, a recent systematic review of quality indicators for end-of-life care for PLwD identified 976 different indicators, of which less than 2% were related to the spirituality domain, that appeared so central for Brazilian participants in this study [63]. Our findings suggest that interventions aimed at increasing the access to choice and control for PLwD in the UK, such as through advance care planning, may help them the most to achieve a good death. In contrast, in Brazil it is likely that interventions that draw on and strengthen their spirituality have the highest potential of being successful at the end of life. At the same time, our results illuminate the experience of ‘suffering’ related to a diagnosis of dementia [64, 65]. In the UK, where personal value seems to be largely defined by the ability to be in control, a disease such as dementia, which fundamentally hinders that ability, represents a major threat to the integrity of the individual and is reflected by the fear of losing one’s identity. In contrast, as choice and control were of much less concern for Brazilian participants, there were no reports of fear of becoming ‘an empty shell’ or even a sense that a diagnosis of dementia influenced how participants thought about their deaths. Instead, Brazilian participants used terms like ‘childlike’, without expressing fear or concern around this.

Our results are also relevant in highlighting and supporting the existence of commonalities among contexts, such as the importance of pain relief and wanting to be close to family but not a burden to them, demonstrating that there are aspects to a good death that are cross-cutting to different population groups. A common issue that has implications for the provision of palliative care and that was reported by few studies involves the importance of previous experiences with the death of others in shaping their wishes and fears towards their own deaths [28]. We argue that clinicians should learn to ask about those experiences and consider that the memories of the death of others are also inhabiting the space where current and future care are provided.

Our findings must not be used for the stereotypisation of PLwD anywhere. Within each culture there is so much space for interindividual variability and subcultures that healthcare professionals must always exercise the principles of cultural humility– i.e., an attitude of openness and genuine curiosity towards the perspectives of patients coupled with the necessary self-vigilance to avoid imposing their own cultural values upon patients and their families – even when interacting with patients from their same ethnic background [66, 67]. Instead, our results should be used to expand the awareness and sensitivity of health and social care professionals around different cultural views on what a good death is for PLwD and what helps or hinders achieving it. Beyond this, our findings also stress the importance of asking PLwD (and by extent any other group of people with a life-limiting condition) what a good death means to them, rather than predominantly relying on the perspectives of professionals and family members. While all groups, including our participants, emphasise the importance of a pain-free death and good pain management, the place/environment of death appears to have more importance for professionals and family members, than PLwD [9, 10, 12, 14]. Instead, PLwD in our study often spoke about ensuring that their death is not traumatic or burdensome for their families, even if this meant prioritising what’s best for the family over their own wishes. This underscores that PLwD can and do talk about what a good death means to them, that their views differ somewhat from those of other stakeholders, and thus that consulting PLwD matters.

Our study has a few limitations. Convenience sampling and pragmatic recruitment strategies used in both countries mean that we interviewed PLwD who felt comfortable to talk about death and that, while our interviewee samples were diverse in terms of demographic and illness characteristics, participants’ geographic location did not cover both countries equitably. Secondly, we were not able to determine the severity of the dementia of our participants. This would have required a clinical assessment or access to medical records, raising issues with ethical clearance and feasibility. Nevertheless, the fact that we only included participants who had capacity to provide informed consent suggests that they had mild or moderate dementia. Thirdly, we did not return transcripts to participants for comment or correction, or perform member checking [68]. While this is increasingly becoming a standard in interview-based studies to enhance credibility and participant engagement, the degree to which it does increase credibility remains contested, and the risks of the process being emotionally taxing for participants, especially when researching sensitive topics, may be substantial [69–72]. Fourthly, sample characteristics also differed between the two countries in terms of participants’ experiences living with dementia (e.g., ethnicity, age, and time since diagnosis). Nevertheless, as is typical to qualitative studies, our aim was not to produce generalizations or comparisons in the statistical sense but to enhance the understanding of a good death for PLwD through comparisons between two different countries. Indeed, transferability in qualitative studies is seen as a collaborative effort between researchers and readers [73, 74]. Based on the researchers’ presentation, readers will evaluate how well the findings resonate with and apply to different contexts that share similar characteristics with the study. Furthermore, despite the fact that a formal Differential Qualitative Analysis (i.e., the qualitative equivalent to subgroup analysis) was beyond the scope of our study, we did not find any clear trends in perceptions around a good death based on demographic characteristics such as age, gender, and level of education (e.g. younger participants did not show different trends in perceptions or preferences compared to their older counterparts across or within countries) [75, 76]. Our study comparing one affluent country in the global north and a middle-income country in the global south also shows the extent to which context and culture matter and challenge attempts to generalise the meaning and measure of a good death [4, 11, 22, 25, 77]. However, we did not compare factors that relate, but are distinct from country of residence. Other factors at country level, such as healthcare systems, public visibility of dementia, levels of dementia stigma, perceptions and expectations around family care, cultural/national identity and others are likely to influence perceptions of a good death and subsequent studies should consider such comparisons, too.

Our study also has strengths. Our cross-cultural comparisons are based on a single interview guide and an equivalent data collection procedure in both countries. Our interview guide may be of further use to researchers in other countries, who wish to explore culturally-appropriate approaches to palliative care and compare them cross-culturally. The analysis strategy also ensured that themes specific to each country, as well as a collaborative cross-cutting thematic analysis between the two countries, are available as interview transcripts were first analysed in the language of the participants and by researchers in the corresponding country and then jointly by the whole research team. Conducting the data collection in each country both before and during the COVID-19 pandemic has further strengthened our findings in showing their applicability at and outside of time periods when globally the public are sensitised to death and dying.

In conclusion, our findings expand the understanding of how culture and context may influence how PLwD define a good death. There is the potential to widen the awareness and sensitivity of health and social care professionals to different patients’ perspectives about what a good death means and what helps or hinders achieving it. Such cross-cultural comparisons offer new opportunities for designing culturally-sensitive approaches to palliative dementia care and challenge global indices of quality of death that are heavily skewed towards the global north and are mostly based on the views of families and healthcare professionals.

## Supplementary Information

Supplementary Material 1. Semi-structured interview guide.

Supplementary Material 2. Background of researchers related to this study.

Supplementary Material 3. Standards for Reporting Qualitative Research (SRQR) checklist.

Supplementary Material 4. COnsolidated criteria for REporting Qualitative research (COREQ) checklist.

## Data Availability

Because we do not have the permission of participants to share the transcribed interviews and to preserve their anonymity, the transcribed interviews are not available for sharing. In case other researchers wish to have access to the anonymised interview transcripts they should contact Rasa Mikelyte (R.Mikelyte@kent.ac.uk) for the UK data and Edison I.O Vidal (edison.vidal@unesp.br) for the Brazilian data while understanding that any access to the transcripts is conditional to the approval by the specific Ethics Review Committee.
