# Supplementary material for: “It’s my life, it’s my choice and I want to say when” vs “A good death is to be on good terms with God”. Comparing the views of people with dementia in the UK and Brazil about a good death: a cross-cultural qualitative study
Source: BMC Palliat Care. 2025 May 16;24:138. doi: 10.1186/s12904-025-01771-w (PMC12082953; doi:10.1186/s12904-025-01771-w)
Supplement: Supplementary file 1 — Supplementary Material 1. Semi-structured interview guide. [file 12904_2025_1771_MOESM1_ESM.docx]

**Appendix 1:** Semi-structured interview guide

| Thank you for agreeing to this interview, we are here to listen to your thoughts on death and dying and what a good death means to you.  This interview is about improving our understanding of what people with dementia see as a good death and dying in light of their diagnosis. With your permission, we will audio record the interviews.  If you feel that you need to stop or leave the room please tell me.  Whatever you tell me will be pseudonymised for the purposes of the study.  At the end of the interview, if you have any issues or comments we will make a note in our ‘issue log’.  The main aim of the interview is to obtain the following information:   - Your perceptions of what is a good death - Your perceptions of what would be a bad death - What kind of things might make a good death? - Later, we will compare what people in the UK think to those in other countries. |
| --- |

1. Firstly, what would a good death mean to you?

*Potential prompts:*

- Do you know anyone who has had what in your opinion is a good death?
  - What was it like? Did the person die from a diagnosed illness? Do you know what it was?

1. Are there specific things that you would wish for as you near death?
2. Are there specific things that you fear for as you near death?
3. What difference – if any – does your dementia diagnosis have on what a good death means to you?
4. Do you feel you know as much as you want to know about what may happen to you as you near death?

5.1. Have you been told what to expect as your dementia progresses? By whom (if at all)?

*Potential prompts:*

- - How important is it to you to know in advance what is likely to happen as you near death?
  - What are your thoughts and feelings about the uncertainties around death and dying?
  - What symptoms do you think people may experience when dying?
    - *Wait for spontaneous response. If the interviewee asks for examples, mention* pain, difficulty breathing, incontinence, inability to chew or swallow, or loss of consciousness.

1. Is it important for you to have control over your death? [*If yes*] What would having control feel like?

*Potential prompts:*

- - What preparations do you think can be made for a person end of life?
  - Have you made any preparations already or are thinking about doing so?
    - E.g. a will, LPA’s, advance care plan, etc.

1. Would you want to be alone or have someone else with you when you die?

*Potential prompts:*

- How important would it be to have family/friends nearby? How can friends and family influence the quality of your death?
- Would you want anyone else there, like a medical professional, a spiritual leader or a pet?
- What kind of role would you like them to have?
- Is it important to you to say goodbye to people? *If so,* when and how would you do it?

1. Where do you think it would be good to die?

*Potential prompts:*

- Do you have a preference for dying in your own home, someone else’s home, hospice or hospital? Why?
- Is there a place you definitely would not like to die? Why?
- If dying in hospital or hospice meant your symptoms (e.g. pain) would be more under control, would that affect your choice of where to die and why?

1. How can health (and social care) providers influence the quality of your death?

*Potential prompts:*

- What about nurses? doctors? paid care workers? Anyone else?

| **To be administered to the participants verbally, *after* the interview.**  Age: ________  Gender: ______________  Type of dementia (if known): ______________  Approximate length of time since diagnosis: ____________  Ethnicity: _______________  Religious affiliation (if any): _______________  Relationship to main informal carer? _______________  Does the carer live with the respondent? Yes No |
| --- |
