# Supplementary material for: “It’s my life, it’s my choice and I want to say when” vs “A good death is to be on good terms with God”. Comparing the views of people with dementia in the UK and Brazil about a good death: a cross-cultural qualitative study
Source: BMC Palliat Care. 2025 May 16;24:138. doi: 10.1186/s12904-025-01771-w (PMC12082953; doi:10.1186/s12904-025-01771-w)
Supplement: Supplementary file 2 — Supplementary Material 2. Background of researchers related to this study. [file 12904_2025_1771_MOESM2_ESM.docx]

**Appendix 2**

**Background of researchers related to this study**

EIOV is a white cisgender male Brazilian geriatrician and palliative care physician in his mid-forties with experience in caring for people living with dementia from diagnosis to the end of life. He has experience in working in a variety of contexts and settings ranging from public and private hospitals to home care, and from the Amazon to the city of São Paulo. Both of his grandmothers died with dementia more than two decades ago. He was not previously known to the participants that he interviewed for this study.

RM (she/her) is a white cisgender woman in her early-to-mid thirties who, despite living and working in the UK for over 15 years has retained a distinguishable eastern european accent. RM’s disciplinary background is in psychology and social policy; she now works in applied health and social care research. RM, who conducted all of the UK-based interviews, was not previously known to any of the participants. RM has also been a part-time dementia care worker for 10+ years, briefly worked in an NHS memory clinic, and has past family experience of dementia.

DO (she/her) is a white nurse researcher from Brazil currently working as an academic in Chile and as consultant for international organizations. Her experience and expertise lies on long-term care, as well as on the psychosocial experiences of people living with dementia and their care partners, including stigma and discrimination. She has experience in both qualitative and qualitative research, and on policy development and evaluation.

KHD is a white British cisgender female and a nurse by professional background with over 45 years’ experience. The majority of this time has been spent in the field of dementia care, in a range of care settings and roles, including hands on nursing, developing practice of other professionals, managerial and finally as a nurse researcher in the charity sector collaborating with a wide range of academics. Her research interests largely focus on palliative and end of life care in dementia, including advance care planning. Her research methodology experience lie within qualitative and mixed methods, and more recently in realist evaluation. She was involved in the development of this research study, in transcribing and analysing data and in writing the final report. She was not known to any of the participants. She has experienced dementia in several family members and friends.

FPR(she/her) is a white cisgender female Brazilian speech and language pathologist specialized in gerontology, palliative care and dysphagia in her early-to-mid-forties with experience in rehabilitating, educating, studying and teaching about people living with dementia from diagnosis to the end of life. He has experience in working in a variety of contexts and settings including public and private care, as community places, primary care settings, nursing homes, hospitals, rehabilitation clinics and home care, public and private hospitals to home care, and from the Northeast to the Southeast of the country. She has been working as SLP for 23 years, 21 of them also as an academic in public universities. One of her grandmothers died with dementia more than one decade ago. She was not previously known to the participants whose interviews she transcribed and/or analyzed for this study.

NLD is a white cisgender woman, cultural and medical anthropologist based at the University of Amsterdam. Her research focuses on ageing, end-of-life care, dementia, and grief. She has extensive experience with ethnographic and qualitative methods and was not previously known to the participants of this study.

AFN is a white cisgender Brazilian psychiatrist in her early thirties. When she joined this research project she was a psychiatry fellow. She has experience in caring for people with mental illnesses including people living with dementia in both in-hospital and ambulatory settings. One of her grandmothers had a dementia diagnosis and passed away 6 years ago. She was not previously known to the participants that she interviewed for this study.

JMV is a white cisgender Brazilian woman and physician in her mid-twenties. When she joined this research project, she was still a medical student and had experience caring for people living with dementia under that role. She was not previously known to the participants that she interviewed for this study.
